# High-fidelity chip delayering using green (515 nm) femtosecond lasers

**DOI:** 10.1038/s41598-026-35091-7

**Published:** 2026-01-20

**Authors:** Mohammad Taghi Mohammadi Anaei, Matthew Maniscalco, Hongbin Choi, Marcus Emanuel, Wesley Roser, Todor Bliznakov, Toni Moore, Adrian Phoulady, Parisa Mahyari, Alexander Blagojevic, Nicholas May, Garth C. Egan, Sina Shahbazmohamadi, Pouya Tavousi

**Affiliations:** 1 University of Connecticut, Storrs, CT USA; 2 The MITRE Corporation, Bedford, MA USA; 3 Tescan Group, Storrs, CT USA

**Keywords:** Femtosecond laser, device delayering, 3D imaging, Design Reconstruction, Multimodality investigation, Engineering, Materials science, Optics and photonics

## Abstract

Accurately reconstructing the metal layers of semiconductor chips is essential for legacy hardware support, design validation, and failure analysis. Conventional methods such as mechanical polishing, chemical etching, and focused ion beam (FIB) delayering, while established, tend to be slow, inconsistent, and resource-intensive—making them less suitable for systematic or scalable workflows. To address these limitations, we developed a streamlined approach combining laser-based delayering with high-resolution multimodality microscopy, offering a more efficient and reproducible alternative. Building on our earlier work with infrared laser delayering, which faces challenges related to selective material interactions and uneven ablation, in this work, we have investigated the use of a green (515 nm) laser. This alternative wavelength offers reduced sensitivity to material variations, allowing for more uniform and controlled removal of chip layers. Through a thorough parameter space exploration and optimization process, we achieved significantly cleaner delayering and exposure of underlying structures. The effectiveness of this method is demonstrated through comparative imaging using confocal microscopy and SEM, as well as material analysis via EDS, all showing notable significant improvements in layer clarity and debris reduction. These results highlight the green laser’s potential as a powerful tool for high-fidelity chip analysis in modern diagnostics and reverse engineering workflows.

## Introduction

The ability to precisely reconstruct internal features within semiconductor devices is foundational to several critical areas in the electronics ecosystem, including legacy system support, reverse engineering, failure diagnostics, and design validation^1,2^. A growing number of legacy systems—spanning aerospace, defense, industrial, and medical sectors—have become non-operational or severely backlogged due to reliance on obsolete integrated circuits (ICs) for which no fabrication data or replacement parts exist^3^. In such cases, design reconstruction through reverse engineering remains the only viable path to restoration. Simultaneously, in contemporary semiconductor manufacturing, the absence of rapid, high-resolution methods for verifying and validating fabricated chips against their intended designs poses a significant risk. Flaws introduced during manufacturing—whether accidental or malicious—can propagate undetected into critical infrastructure. Without efficient tools to compare built structures to their original layouts, design-rule violations, latent defects, or hardware Trojans may remain hidden until catastrophic failure occurs. Furthermore, failure analysis itself is often time-consuming, delayed by the lack of precise and scalable layer-by-layer inspection techniques^4^.

Among the IC’s internal structures, metal interconnect layers are especially vital, as they define the electrical connectivity between devices and subsystems. Accurately exposing and analyzing these layers is essential for understanding circuit function, identifying anomalies, and ensuring long-term reliability^5^.

As chip architectures become increasingly complex and component dimensions continue to shrink, traditional methods for exposing internal layers face growing limitations. Common techniques such as mechanical polishing and chemical etching are inherently imprecise and inconsistent, often leading to layer damage or incomplete material removal. These methods are also labor-intensive and difficult to automate, making them impractical for large-scale or iterative use. Focused ion beam (FIB) delayering, while highly precise and widely adopted in failure analysis, suffers from inherently slow material removal rates, making it time-consuming and limiting its applicability to large-area reconstructions or high-throughput environments^6–8^.

Non-destructive imaging alternatives—such as lab X-ray systems—offer limited resolution, often insufficient to resolve microscale interconnects. Though synchrotron-based X-ray computed tomography can achieve the required resolution, such facilities are costly and inaccessible to most industrial and academic labs, especially for routine diagnostics^9^. In response to the limitations of traditional delayering techniques, laser-based approaches have emerged as a compelling alternative, offering high-speed, non-contact, and scalable layer removal. Ultrafast pulsed lasers, in particular, enable precise ablation with minimal thermal diffusion, thus preserving the integrity of underlying microstructures^6–8,10,11^. In our earlier studies, we employed infrared (IR) ultrashort pulsed lasers to delayer semiconductor chips^12^. While successful in removing material, this approach was constrained by the wavelength-dependent absorption behavior of different chip constituents—metals, dielectrics, and semiconductors—resulting in non-uniform ablation, debris accumulation, and incomplete or damaged layer exposure. These inconsistencies posed challenges for high-fidelity imaging and subsequent analysis.

In this work, we demonstrate a significantly improved delayering methodology using a 515nm green (shorter-wavelength) ultrashort pulsed laser. At reduced wavelengths, nonlinear absorption mechanisms become more prominent^13^ and the laser energy couples more uniformly across diverse materials, thereby reducing sensitivity to variations in optical and thermal properties. This shift leads to more predictable and confined ablation behavior, allowing for cleaner material removal and better preservation of structural features. We conducted a comprehensive study of key processing parameters—including pulse energy, repetition rate, scan speed, and focus depth—to optimize the delayering process.

The performance of the green laser delayering approach was rigorously validated through high-resolution confocal microscopy, scanning electron microscopy (SEM), and energy-dispersive X-ray spectroscopy (EDS), all of which revealed substantial improvements over IR-based delayering—including enhanced layer uniformity, cleaner interfaces, reduced debris, and significantly improved visibility of metal interconnects and adjacent dielectric structures. These results demonstrate the green laser’s effectiveness as a precise, repeatable, and material-tolerant solution for semiconductor layer reconstruction.

By addressing the shortcomings of both traditional and prior laser-based approaches, this work establishes green laser delayering as a highly effective and scalable technique for semiconductor analysis. The method has broad applicability in modern chip development and diagnostics, offering a robust toolset for reverse engineering, failure analysis, and validation of both legacy and cutting-edge semiconductor technologies.

## Materials and methods

### Test sample

**Fig. 1:**
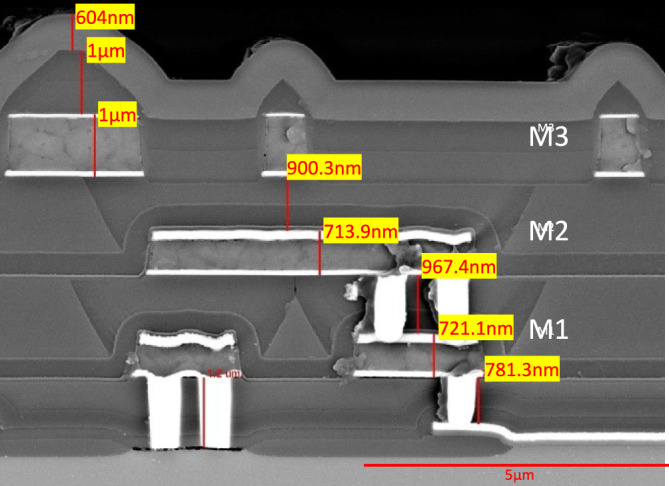
Cross section of microprocessor integrated circuit used in this work, demonstrating the three metal layers.

This research centers on the analysis of a semiconductor microprocessor chip, which features three essential metal layers integral to its operation and electrical performance. A cross section for the chip is demonstrated in Fig. 1. Based on measurements, the total thickness of the IC from the polysilicon to the top of the protective nitride coating is 7.1 µm while the polysilicon contacts (a signifier of technology node) are ~930 nm wide and 470 nm tall. The chips were purchased in bare die format and further included an ~6 µm thick polymeric coating.

### Methodological framework

Expanding upon our earlier findings^7^, where we demonstrated the feasibility of sequential laser delayering—optionally combined with focused ion beam (FIB) polishing—and high-resolution imaging for three-dimensional chip reconstruction, this study extends the methodology to full-chip analysis to evaluate its scalability and robustness. In particular, we apply and optimize a green ultrashort pulsed laser for delayering and systematically compare its performance to that of previously used infrared lasers, highlighting improvements in layer uniformity and feature visibility across an entire device.

### Region-specific laser delayering strategy

**Fig. 2:**
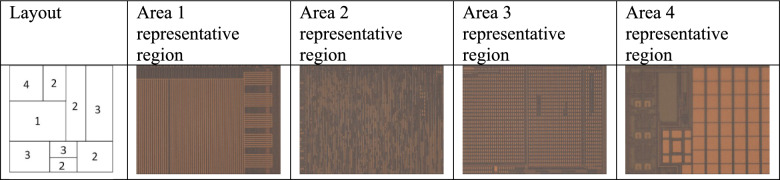
Four distinct types of regions in terms of architecture were identified; the lasering recipe was optimized for each.

A key challenge in delayering arises from architectural variability across the chip. Different sections exhibit diverse structural and material compositions, necessitating individualized tuning of laser parameters. To address this, we identified four distinct region types on the chip that are shown in Fig 2, each characterized by distinct feature sizes, dielectric to metal ratios, and periodicity patterns:

- *Area 1 – large-pitch metal interconnects*: Highly regular line-and-space patterns with ~2 µm-wide metallic traces and roughly 50 % metal coverage. This geometry represents coarse-pitch interconnect routing layers.
- *Area 2 – intermediate-density interconnects*: Networks of ~1 µm-wide irregular metal lines with an estimated ~40 % metal fraction, reflecting intermediate routing layers with locally dense, nonuniform connectivity.
- *Area 3 – dense arrays*: Regular grids of ~2 µm metallic vias with ~ 4 µm pitch and 60–70 % metallic fill, characteristic of memory-cell or via-array regions.
- *Area 4 – large pad and mixed zones*: Coarse ~20–30 µm metal pads separated by wide dielectric trenches, typical of bond pad regions transitioning into fine interconnect areas, as well as ~1 µm-wide metal lines.

Defining these categories allowed for laser settings to be optimized to the specific material properties encountered, enabling precise and material-sensitive ablation. This adaptive strategy enhances both the accuracy and consistency of the delayering process, ensuring that structural fidelity is preserved throughout the imaging and reconstruction phases.

### Optimization procedure

To identify the optimal parameters, we first organized the key variables that most significantly affect the ablation process, including cutting depth and surface quality. These variables include but are not limited to laser power, number of cycles, pulse width, burst mode, and scanning speed. To simplify the process, we held all parameters constant except one, allowing us to observe the effect of each variable on ablation quality individually. It is important to note that multiple configurations can yield similar results; therefore, optimization focused not only on outcome quality but also on minimizing runtime.

### Laser system configuration

Delayering was carried out with a Tescan FemtoChisel tool that uses a femtosecond laser source operating at a wavelength of 515 nm and 1030 nm with a maximum output power of 11.3 to 20 W. The laser produces ultrashort pulses of sub-300 femtoseconds (fundamental pulse width) and supports a broad range of repetition rates, from single pulses to up to 1.1 MHz. The beam was directed through a SCANLAB intelliSCANse 20 galvanometer scanner. The laser beam was focused onto the chip surface via a telecentric F-Theta lens with a 70 mm focal length for IR and 75 mm focal length for Green.

### Imaging procedure

Post-delayering imaging was conducted using a Keyence VK-X3100 laser-scanning confocal microscope. The system employed objective lenses with 20X, 50X, and 150X magnifications to capture detailed surface topographies. Images were acquired at a resolution of 1024 × 768 pixels. Based on the calibrated fields of view, the effective pixel sizes were: 20X = 1.37 × 1.37 µm/pixel, 50X = 0.27 × 0.27 µm/pixel, and 150X = 0.094 × 0.094 µm/pixel.

This configuration allowed for high-fidelity visualization of fine structural details essential to accurate reconstruction. The ZEISS Crossbeam 340 was used to capture the SEM images and Microscopy/Energy Dispersive X-ray Spectroscopy (EDS). SEM imaging was conducted under high-vacuum conditions using both secondary electron (SE) and backscattered electron (BSE) detectors to capture detailed surface topography and compositional contrast. Accelerating voltages ranged from 5 kV to 20 kV depending on the imaging requirements and material composition, with working distances typically maintained between 5–10 mm for optimal resolution. Magnifications varied from 500X to over 100kX based on the feature of interest.

For elemental characterization, EDS analysis was carried out using an integrated Oxford Instruments X-MaxN 80 mm^2^ silicon drift detector (SDD), which provides high count rates and excellent energy resolution for both light and heavy elements. The system was operated at 15–20 kV to ensure sufficient X-ray excitation, and both point spectra and 2D elemental maps were acquired. Acquisition times ranged from 30 to 120 seconds, depending on the signal strength and area analyzed. Elemental mapping was performed to investigate material composition, contamination, and interface layers across delayered semiconductor structures. Calibration was done using certified reference materials to ensure accuracy.

To mitigate charging effects during analysis of non-conductive or partially delayered surfaces, samples were sputter-coated with a thin conductive layer (e.g., gold or platinum) when necessary. The combined SEM/EDS analysis provided high-resolution structural and chemical information, enabling a comprehensive assessment of each layer’s morphology and composition and facilitating defect localization across complex chip architectures.

## Results

### Parameter optimization

In all cases, these parameters were systematically tuned through a dedicated optimization phase before being applied to the chip-level delayering experiments reported here.

We began with sub-300 fs pulse durations, single-burst operation, 25 m/s scan speeds as the initial parameters, based on our prior experience with chip delayering. The first set of tests focused on varying the pulse energy in the range of 0.2 µJ to 4 µJ for a single scan cycle. Results were recorded in terms of runtime and surface quality after ablation. Laser parameters were optimized by tuning the number of cycles and power to mitigate ablation nonuniformity arising from the coexistence of metal and non-metal regions on the chip.

In the second round of tests, the energy per pulse was held constant within this range while the number of cycles was varied from single to multiple passes to evaluate the effect of repetition on surface quality. These experiments were performed across different power levels within the same range.

For the second layer of the chip, a similar optimization approach was used. Due to the ~µm-scale spacing between layers and the architectural complexity of the chip, it was necessary to deliver energy with controlled penetration. After exploring different burst modes (from femtosecond to picosecond scales), repetition rates spanning 10–200 kHz, and scan speeds from tens up to several hundred mm/s, we found that pulse width strongly influenced the surface response. Increasing the pulse width required higher energy for effective ablation. In these tests, pulse energies extended from 5–15 µJ, pulse widths ranged from 300-10,000 fs, and the number of cycles varied from 1–10.

Cleaning was found to be critical: without it, debris accumulated on the surface, whereas introducing CO₂ and N₂ gas blowing during ablation significantly improved surface quality by removing particles^10^.

Note that, while the use of directed CO₂ and N₂ gas flow during ablation follows our general gas-assist approach described in Ref^10^., the pulse energy, repetition rate, and scan speeds were independently tuned for the present study.

To clarify the influence of individual parameters, we summarize the main qualitative trends observed in our experiments below:

- *Pulse energy*: Increasing pulse energy at fixed repetition rate and scan speed improves dielectric removal, but beyond an optimum leads to metal edge rounding.
- *Repetition rate*: Raising the repetition rate at constant fluence and scan speed increases throughput, but at the cost of compromising cutting trench quality beyond an optimum value.
- *Scan speed*: Increasing the scan speed enables smoother and more uniform removal at high pulse energies.
  Number of cycles (passes): Increasing the number of cycles increases the cutting depth. For, best metal removal, large number of cycles must be used with small energy per pulse.
- *Pulse duration*: Using longer pulses improves the removal uniformity (as opposed to shorter pulses with significant impact on dielectric while having minimal effect on metal). Also, it is observed that longer pulses improve vertical resolution by reducing the effective penetration depth of the laser pulse.

### Removal of passivation layer to expose the topmost metal layer (M3)

The first step in the delayering procedure focused on eliminating the passivation layer that protected the uppermost metal layer (M3) of the chip. Based on cross-sectional images (Fig. 1), the coating of the bare die format chips included approximately 6 µm of polymer dielectric, 0.6 µm of nitride, and 1 µm of oxide. In this study, in addition to the previously explored IR laser, we utilized a laser system with a wavelength (515 nm) in visible green portion of the electromagnetic spectrum. Green lasers are known for their high absorption rates in a wide range of materials, especially metals and certain dielectrics, making them particularly suitable for precision micromachining and surface modification applications. Their shorter wavelength compared to infrared lasers further enables better focusability, resulting in smaller spot sizes and more controlled material interaction, which is critical for applications demanding fine detail and minimal thermal damage^14^.

Two approaches to implementing the green laser were explored. In the first, the green laser was employed as a post-processing tool used specifically to polish the surface after initial milling with an infrared laser. Use of green laser significantly improved surface quality by reducing roughness and removing residual material, especially when compared with additional IR steps. In the second approach, all material removal was performed exclusively with the green laser, allowing us to take advantage of its precision and enhanced surface finish throughout the entire delayering workflow. For both approaches, a single parameter set—determined from the optimization process described above—was used over the entirety of the chip surface rather than needing to tailor parameters based on the areas shown in Fig 2. The results for these two approaches are demonstrated in the following sections.

### Approach 1: using green laser as a postprocessing tool

In the first approach, the process started by using an infrared (IR) laser to remove material and expose the target layer beneath. The IR laser is well-suited for high-throughput bulk material removal due to its deeper penetration and strong interaction with a wide range of semiconductor material^15^.

Three distinct regions on the chip (i.e. areas 1, 2 and 3 in Fig. 1) were selected to test the laser’s performance under varying structural and material conditions across the chip surface. IR laser was applied to remove the passivation layer that protects the uppermost metal layer (M3). The delayering process was carefully controlled to ensure consistent exposure of the underlying features while preserving their structural integrity.

**Table 1:**
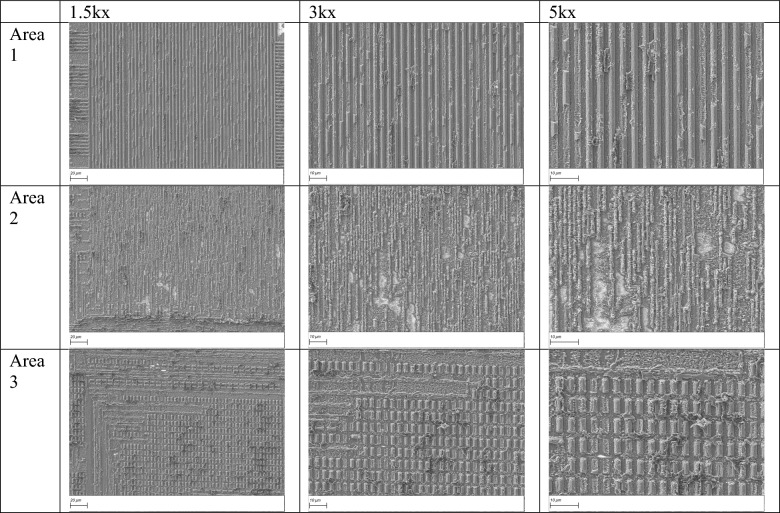
SEM imaging of the first metal layer, after removal of the passivation layer with IR laser, for areas 1, 2 and 3 at three different magnifications.

Table 1 provides the scanning electron microscope (SEM) images for each of the three regions after the initial IR step, at different magnifications.

As shown in Table 1, although the IR laser effectively removes the passivation layer and exposes the top metal layer (M3), the resulting surface remains unpolished and uneven, leading to suboptimal image quality for design reconstruction purposes. To address this, we employ a green laser (515 nm) in the subsequent step to polish the surfaces previously exposed by the IR laser. The shorter wavelength and superior absorption in materials commonly found in IC chips allow for greater precision and improved surface quality compared to infrared light. Formatting...

The green laser is used to perform a fine polishing process, which helps in reducing surface roughness, removing residual debris, and enhancing the clarity of the exposed features. This step is critical for improving the quality of subsequent imaging—especially under laser confocal or electron microscopy—by producing cleaner and more uniform surfaces. By applying the green laser as a post-processing tool, we effectively refine the delayered regions, ensuring minimal thermal damage and maintaining the structural fidelity of the metal traces. This approach enhances the resolution and reliability of the reconstruction process that follows. Table 2 compares SEM images of the surfaces before and after the application of the green laser for areas 1, 2, and 3, shown at three different magnifications.

**Table 2:**
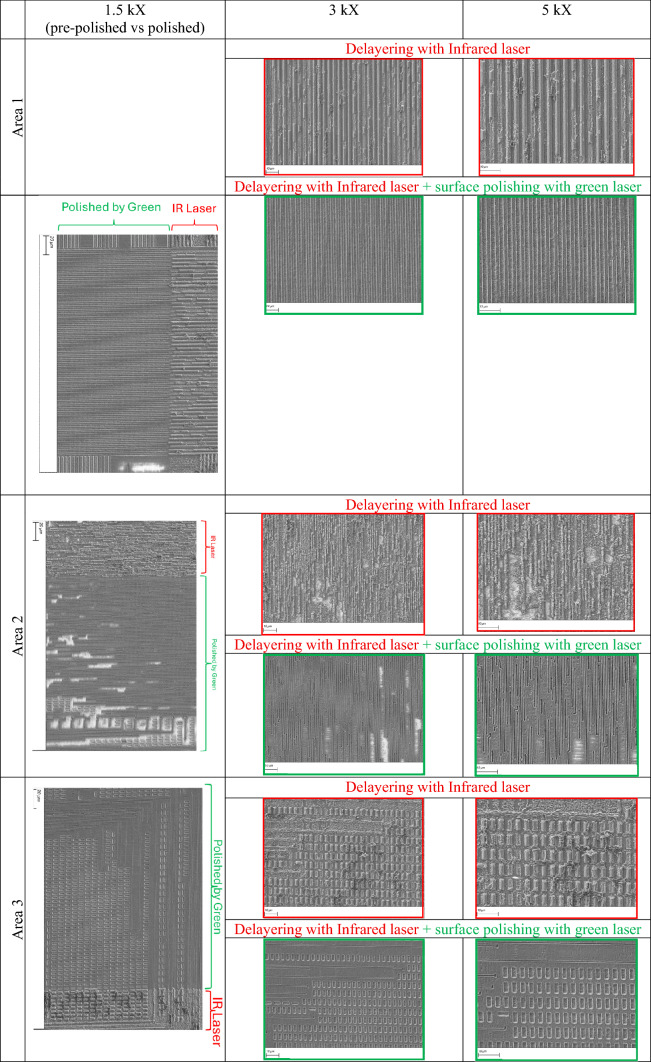
SEM images of the surfaces pre- and post-polishing with green laser, for areas 1, 2, and 3.

#### Rationale behind using IR laser for bulk removal

In our workflow, the IR laser can potentially be better suited for high-throughput bulk removal for two practical reasons. First, the 515 nm “green” beam used here is generated by frequency doubling of the 1030 nm fundamental. This conversion is not lossless and inherently limits the available average power at 515 nm to a fraction of what is available at the IR fundamental. For bulk semiconductor removal, where throughput is primarily power-limited rather than resolution-limited, it is therefore more efficient to use the IR beam for the heavy material-removal steps and reserve the green beam for the final, high-fidelity polishing passes. Second, at 1030 nm the optical penetration depth in silicon-rich and passivation stacks is generally larger, promoting more volumetric energy coupling and faster bulk ablation, whereas the shorter-wavelength 515 nm beam enables tighter focusing and shallower, more controlled removal at interfaces. By the same reasoning, an IR source used at its fundamental wavelength could, in principle, offer higher available pulse energy than a frequency-doubled green beam from the same laser head, but would trade off some of the focusing and multi-material coupling advantages that make 515 nm attractive for precision delayering.

### Approach 2: direct delayering with green laser

In the second approach, we exclusively adopted the green laser (515 nm) for both removing the passivation layer and polishing the M3 metal layer. By eliminating the need for an initial IR laser milling step, this approach streamlines the delayering process while still delivering high precision and surface clarity. The result is a clean exposure of the underlying metal features, ready for high-resolution imaging. This approach demonstrates the potential of the green laser system to serve as an all-in-one tool for both material removal and surface preparation, simplifying the workflow and reducing processing time without compromising the quality of the exposed structures. Table 3 presents detailed optical and SEM images of various regions of the exposed topmost metal layer (M3), captured at different magnifications. Each area of the chip exhibits distinct structural characteristics, and the delayering results shown in Table 3 validate the effectiveness of our region-specific laser parameter tuning. The images highlight the consistency and clarity of metal trace exposure achieved across varying material compositions and surface geometries.

**Table 3:**
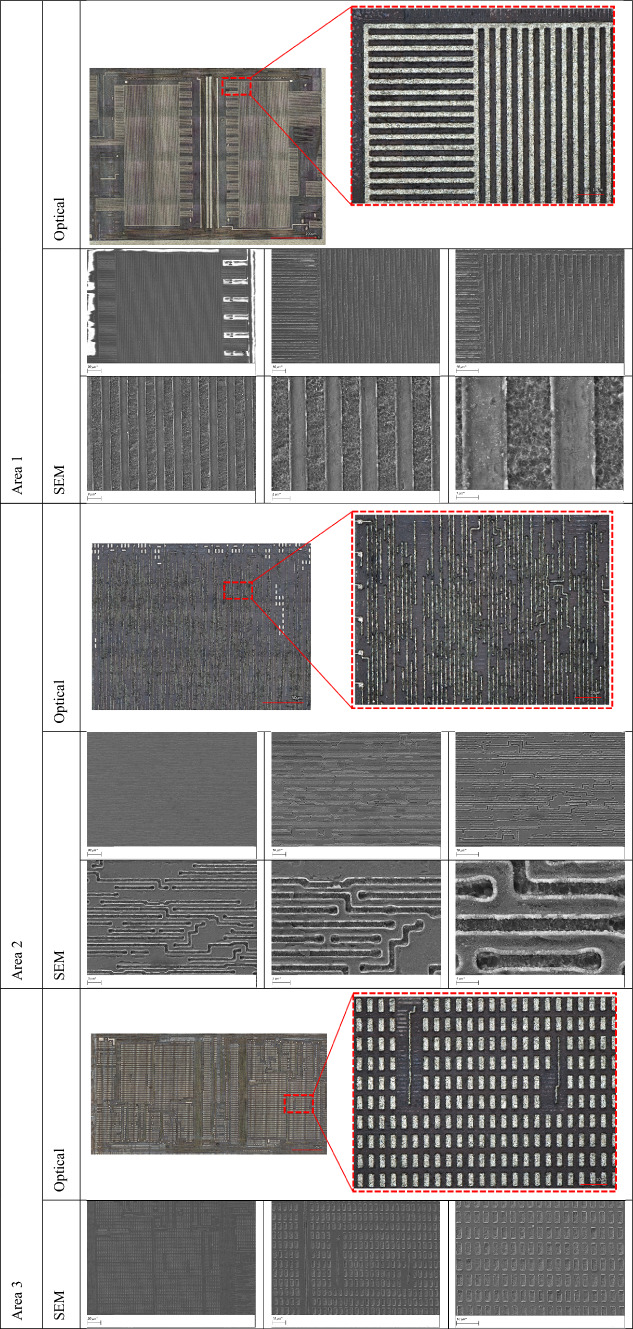

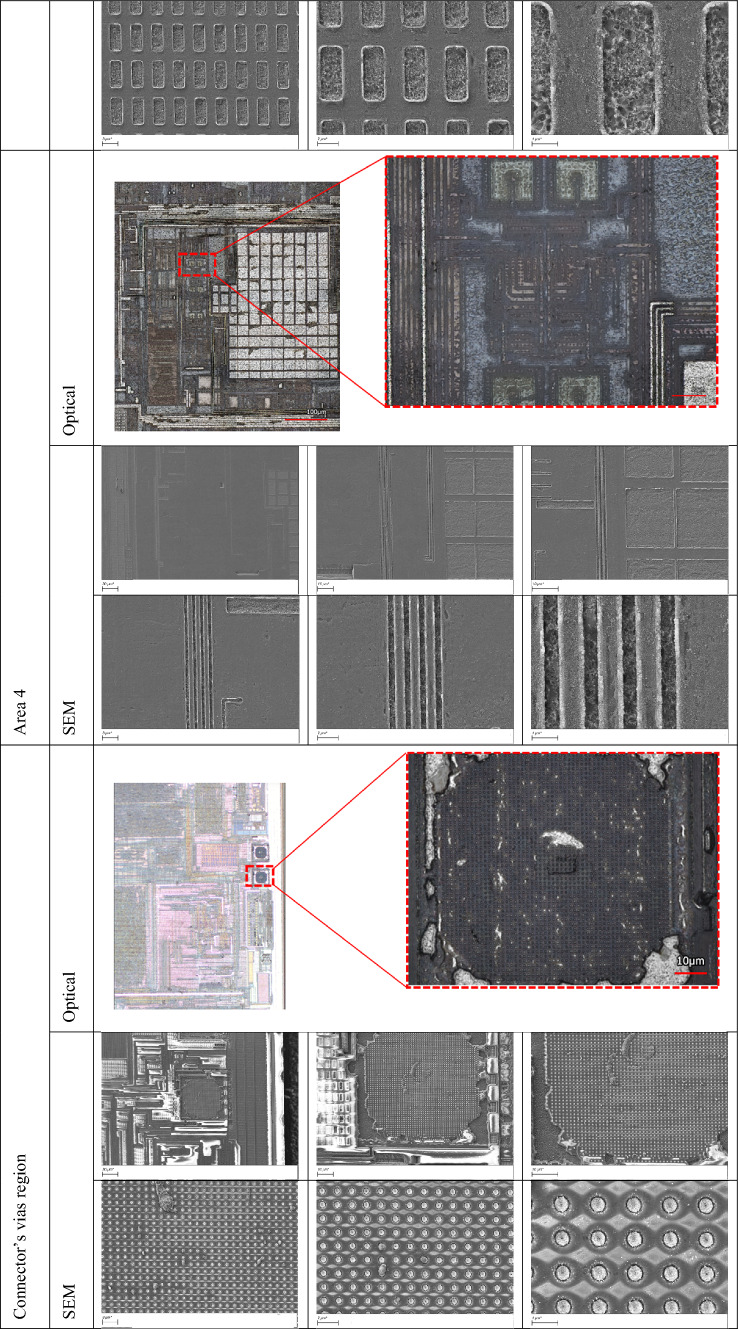
Region-specific optical and SEM characterization of the topmost metal layer (M3).

### Comparison of approach 1 and approach 2

Both strategies yielded good results in terms of surface cleanliness and represent viable approaches to delayering. However the fact that a combined IR–green approach requires the changing of optics or a significantly more complex laser system, make the green only approach much preferable.

### Material characterization of M3 exposed regions using energy dispersive X-ray spectroscopy

Energy Dispersive X-ray Spectroscopy (EDS) was used in conjunction with SEM to investigate the material composition of the exposed chip regions. This integrated analytical technique allows for both high-resolution surface imaging and elemental analysis within the same platform. EDS works by detecting characteristic X-rays emitted from the specimen when it is bombarded with a focused electron beam. These X-rays are unique to each element, enabling qualitative and semi-quantitative identification of the material composition at specific locations on the sample. In this work, EDS was instrumental in confirming the elemental makeup of the metal traces and surrounding materials in the delayered chip regions. By correlating compositional data with structural features observed through SEM, we were able to validate the success of our delayering process and assess the integrity of exposed layers with greater confidence. Table 4 provides the EDS data collected for the different regions of the exposed M3 layer.

**Table 4:**
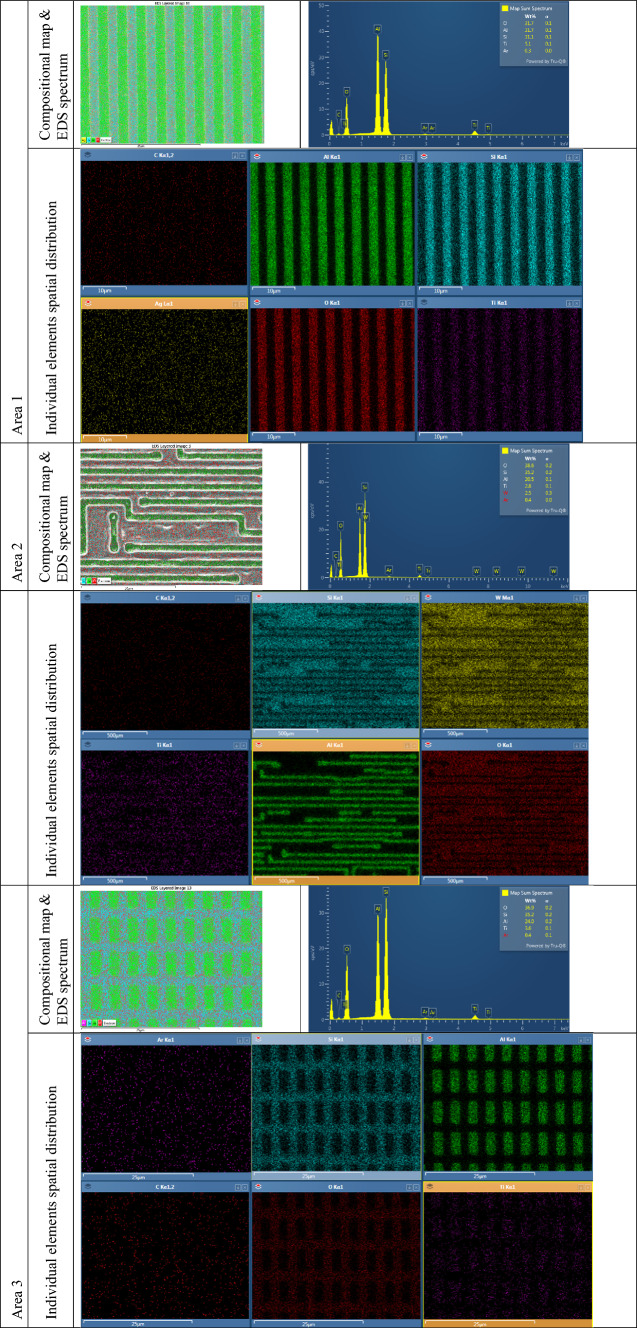

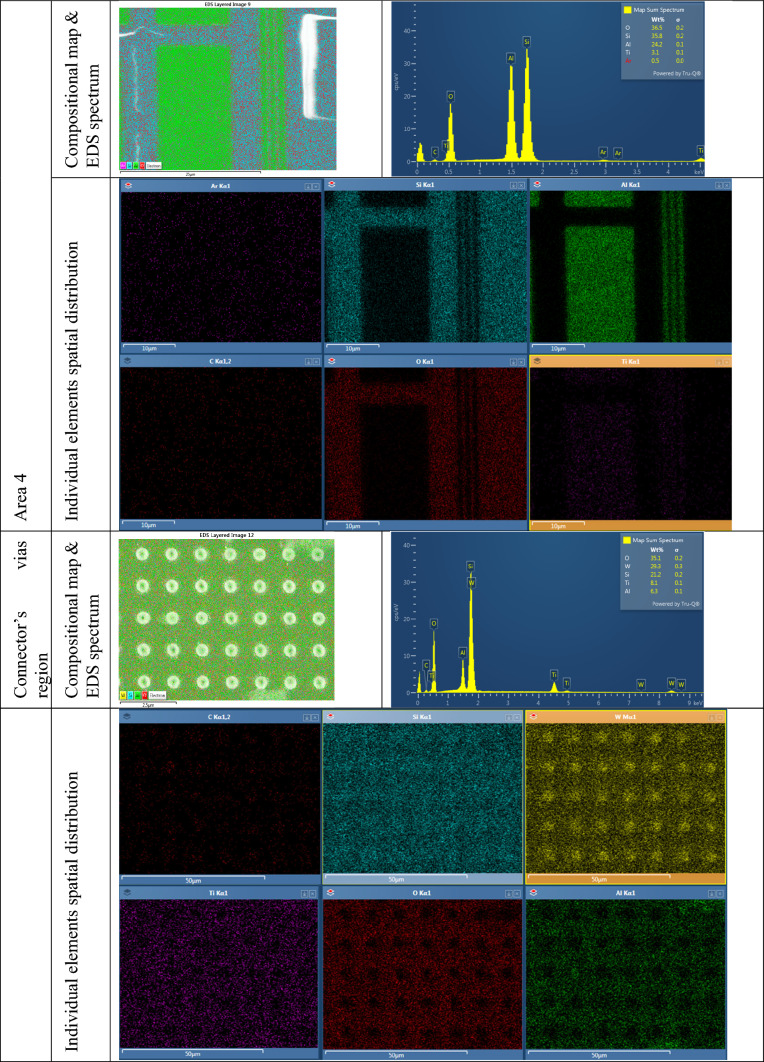
EDS data collected for the different regions of the exposed M3 layer.

### Removal of M3 to expose M2

In this section, we present the second metal layer of the chip, successfully revealed through our laser-based delayering process. This layer was examined across the four previously defined regions (see Fig. 2), each exhibiting distinct structural and material characteristics.

Three-dimensional reconstructions from confocal microscopy and cross-sectional measurements revealed that the vertical spacing between the first and second metal layers is approximately 1 µm. This narrow interlayer distance poses a significant challenge, as it demands high-precision material removal to fully eliminate the first metal layer while preserving the integrity of the second. Accurate sub-micron depth control is therefore essential.

To compare the performance of infrared and green lasers, the entire delayering and imaging procedure was conducted independently using both an IR laser and a green laser. This allowed for a systematic evaluation of each laser’s performance in terms of depth accuracy, surface cleanliness, and preservation of fine features. Consistent with results from the topmost metal layer (M3), the green laser again demonstrated superior performance, producing cleaner exposures.

Successful exposure and imaging of the second metal layer (M2) were achieved in regions 1 and 3 (see Fig. 1). However, delayering in regions 2 and 4 proved more challenging due to localized variations in material and geometry, occasionally resulting in partial or complete removal of the M2 layer, thereby limiting imaging quality in those regions. The results are presented in Table 5.

**Table 5:**
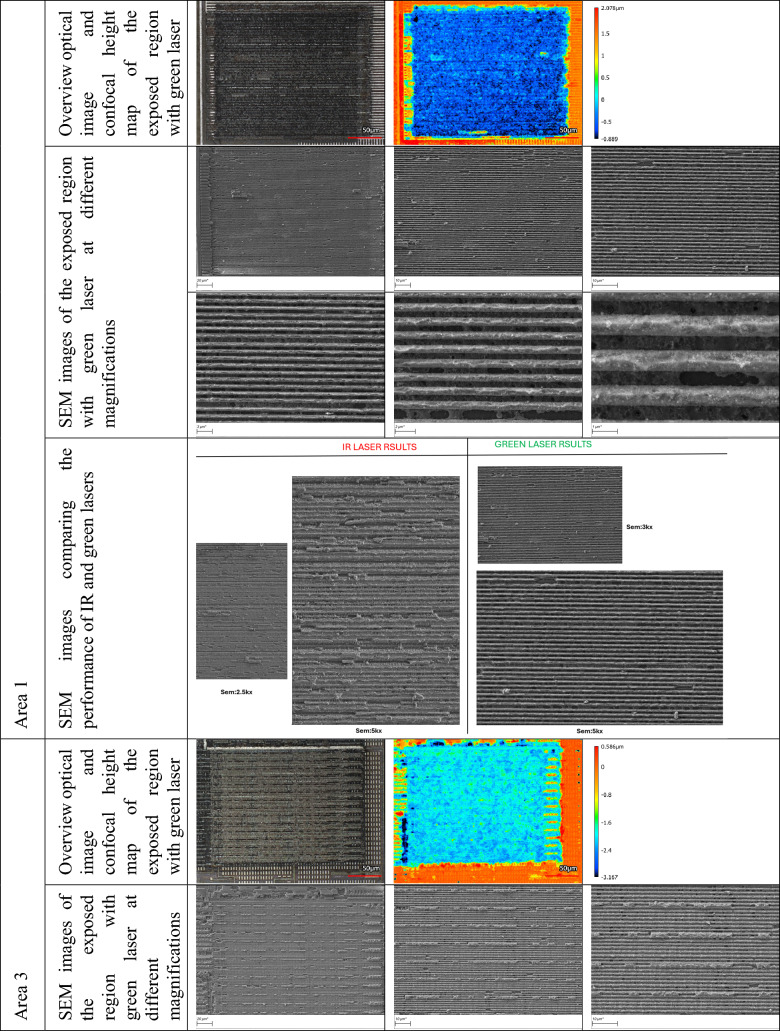

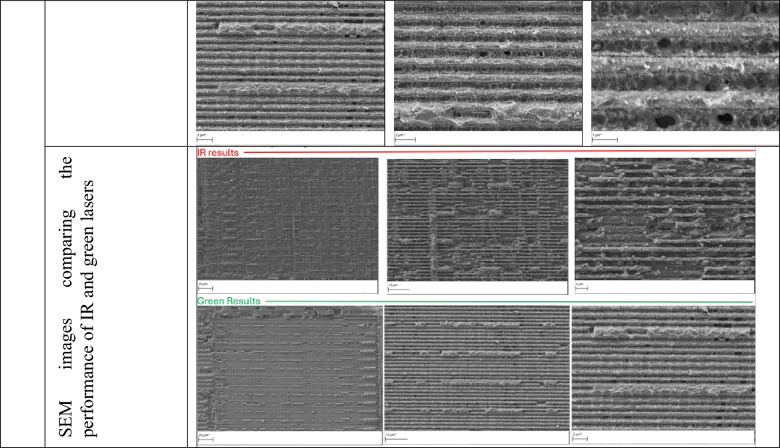
Green laser exposure and imaging of the second metal layer (M2) in regions 1 and 3.

### Extent to which IR laser processing can replicate green laser results

A key question is how closely infrared (IR) laser processing can reproduce the outcomes achieved with green lasers, particularly in terms of surface cleanliness. As shown in Table 6, increasing the number of processing cycles with the IR laser does lead to improved cleanliness, and in certain regions (areas 1, 2 and 3 of the M3 layer, and areas 1 and 3 of layer M2) where the features are relatively larger, the results approach (but do not match) those obtained with green laser processing. Nevertheless, this apparent improvement does not reflect a true optimization of the IR process, but rather the effect of repeated cycling. Importantly, the enhanced cleanliness achieved in this way comes with a significant drawback: the additional cycles result in the removal of metallic material (as clearly evident in the zoomed in image of M3, Areas 2 and 3 – See Table 2). By contrast, the green laser can achieve comparable or superior surface cleanliness while preserving the underlying metal, making it the more advantageous choice for applications where material retention is essential.

**Table 6:**
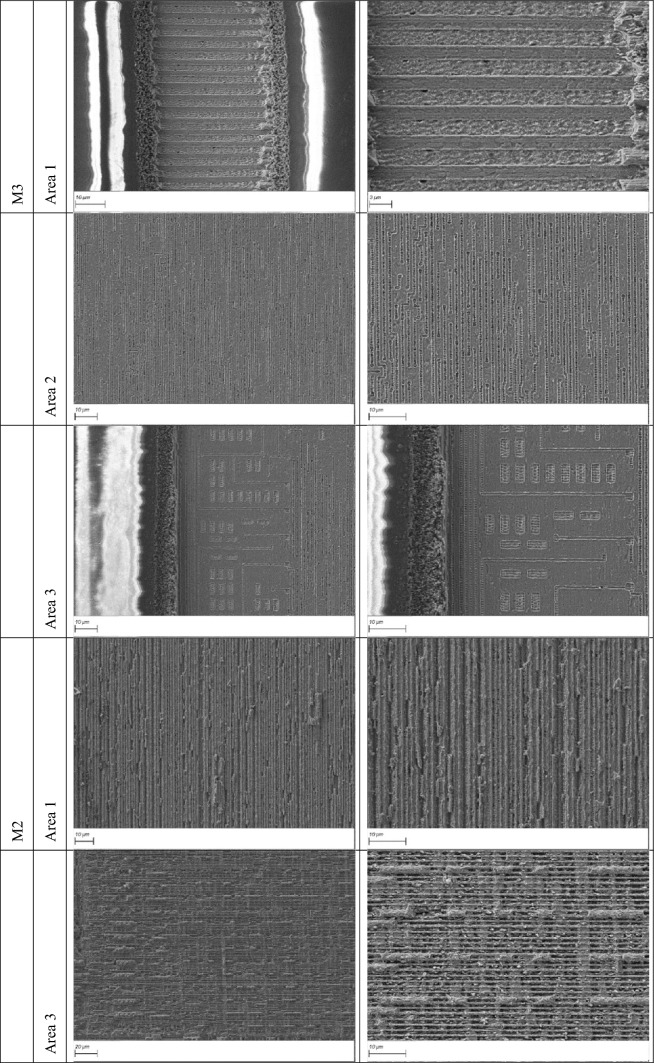
Effect of increased processing cycles on IR laser surface cleanliness.

## Discussion

This study demonstrates the substantial advantages of using a green femtosecond laser over an infrared counterpart for semiconductor chip delayering. The shorter wavelength of the green laser enables more uniform energy absorption across diverse chip materials and tighter focusing, resulting in significantly improved delayering quality. Our experiments showed cleaner exposure of metal layers, reduced debris, smoother surfaces, and improved feature visibility when compared to IR-based delayering.

At the same time, our results highlight nuances in how IR processing can be used. When additional cycles were applied, IR lasers could produce surfaces that were cleaner and, in regions with relatively larger features, the results approached (but did not match) those obtained with green lasers (Table 6). However, this improvement came at the expense of metallic material loss, underscoring a limitation: IR processing can mimic some of the benefits of green lasers, but only by sacrificing layer preservation. This distinction reinforces the superior capability of the green laser to combine cleanliness with material integrity.

These tradeoffs were also evident when green lasers were used in hybrid strategies. In one approach, the IR laser performed bulk removal, followed by green laser polishing as a final step. In another, the green laser was used exclusively for both removal and finishing. Both methods yielded good surface cleanliness, but the IR–green combination again led to metal loss similar to that seen with multi-cycle IR processing. In contrast, the all-green approach delivered comparable cleanliness without compromising metallic layers, making it the more effective strategy for applications where preservation of delicate structures is critical.

Despite these advantages, limitations remain for green-only processing. Even with careful parameter tuning, certain regions—particularly those with complex geometries or variable material compositions—still posed challenges. In some cases, the green laser failed to fully expose target layers without introducing damage or unevenness. These observations raise an important question: Are the observed limitations intrinsic to the green laser technique, or can they be overcome with further optimization or enhancement of the process?

Answering this question requires a deeper and more systematic exploration of the high-dimensional laser parameter space. Additional refinements—potentially through automated or AI-guided tuning strategies—may reveal configurations that further improve uniformity and precision across all chip regions. If such improvements prove insufficient, it may suggest that the green laser, while highly capable, has reached the practical limits of its delayering fidelity.

In such cases, other hybrid approaches become particularly valuable. One effective strategy we have previously demonstrated^12^ involves using the laser for precise, high-throughput bulk material removal, followed by Focused Ion Beam (FIB) polishing to refine critical surfaces. This combination leverages the speed and scalability of laser machining with the unmatched precision of FIB at nanometer-scale feature levels. Another promising direction lies in exploring higher harmonics of the laser, such as ultraviolet (UV) or deep ultraviolet (DUV) wavelengths. These shorter wavelengths can offer even finer spot sizes and improved interaction with smaller features and more challenging materials, potentially enabling higher-resolution and more selective delayering. While higher harmonics introduce challenges related to beam delivery and energy coupling efficiency, their potential to enhance ablation precision—especially for advanced technology nodes—merits rigorous investigation.

Alternatively, improvements may lie in adapting the reconstruction techniques themselves. For example, our prior work^16^ has shown that three-dimensional reconstruction from non-planar or topographically uneven surfaces—produced by laser delayering—can still yield accurate structural information. By embracing rather than avoiding the natural topography left by the laser, and using confocal or SEM-based 3D imaging pipelines, we can bypass the requirement for perfectly flat layers in the reconstruction process. A further strategy involves cross-sectional imaging, where the chip is sectioned perpendicular to the surface rather than layer-by-layer from the top down. Although this approach demands more imaging and stacking effort, it yields inherently cleaner exposure of structural features, especially when combined with advanced segmentation and registration algorithms.

## Conclusion

This work demonstrates a significant advancement in semiconductor chip delayering through the use of a green femtosecond laser, offering substantial improvements over the infrared-based method. By systematically optimizing processing parameters and tailoring them to region-specific material characteristics, we achieved enhanced layer exposure, improved surface quality, and reduced debris—critical factors for accurate design reconstruction and high-resolution imaging.

Both hybrid workflows—using green laser polishing after infrared ablation—and fully green laser-based approaches were evaluated. Results show that the green laser not only improves surface finish when used as a post-processing tool but is also effective as a standalone method for precise, high-fidelity delayering. Imaging and elemental analysis across multiple chip regions confirmed the green laser’s capability to deliver consistent, high-quality results suitable for reverse engineering, failure analysis, and device validation.

While some variability remains in more complex regions of the chip, these cases highlight opportunities for continued refinement rather than fundamental limitations. Further exploration of process parameters, adaptive control strategies, and potential integration with complementary techniques (such as FIB polishing or advanced 3D reconstruction methods) can help extend the approach to even more challenging scenarios. Additionally, the use of higher harmonic wavelengths, such as UV or DUV, may offer further gains in precision and material selectivity, and future work will systematically explore these wavelengths within the same comparative framework used here for 515 nm and 1030 nm to assess the trade-offs among fidelity, robustness, and throughput.

Overall, green femtosecond laser delayering emerges as a robust, scalable, and versatile technique that holds great promise for next-generation semiconductor analysis workflows, supporting both legacy systems and advanced microelectronic technologies.

## Data Availability

All data generated or analyzed during this study are included in this published article.
